# Recent advances in ß-carotene: Biological activities, therapeutic potential, and delivery strategies

**DOI:** 10.17179/excli2026-9677

**Published:** 2026-09-07

**Authors:** Chanung Park, Jae Kwang Kim, Sang Un Park

**Affiliations:** 1 Department of Crop Science, Chungnam National University, 99 Daehak-ro, Yuseong-gu, Daejeon 34134, Republic of Korea; 2 Division of Life Sciences and Convergence Research Center for Insect Vectors, College of Life Sciences and Bioengineering, Incheon National University, Incheon 22012, Republic of Korea; 3 Department of Smart Agricultural Systems, Graduate School, Chungnam National University, Daejeon 34134, Republic of Korea

## ⁯⁯⁯⁯

Carotenoids are naturally occurring pigments that are abundant in a wide range of photosynthetic organisms, including algae, cyanobacteria, and higher plants. In photosynthetic tissues, they contribute to light harvesting and photoprotection, while their nutritional and potential health-promoting properties have also attracted considerable scientific interest (Mezzomo and Ferreira, 2016[6]). Carotenoids also confer yellow, orange, and red colours on flowers and fruits, thereby contributing to pollinator attraction and seed dispersal (Maoka, 2020[5]). Because most animals cannot synthesise carotenoids de novo, they obtain them primarily from the diet (Rodriguez-Concepcion et al., 2018[7]).

β-Carotene is a common dietary carotenoid and a major provitamin A carotenoid. In humans, approximately 17-45 % of ingested β-carotene may reach the peripheral circulation intact, indicating that a substantial proportion can be absorbed without conversion to vitamin A (Shete and Quadro, 2013[8]). Major dietary sources include green leafy vegetables and orange- or yellow-coloured fruits and vegetables such as mango, carrots, spinach, pumpkins and broccoli (Mezzomo and Ferreira, 2016[6]).

β-Carotene has been investigated in relation to cardiovascular disease, cancer, and ocular health, owing partly to its antioxidant properties and its role as a vitamin A precursor (Brambilla et al., 2008[1]; Hamilton, 2007[2]; Jeon et al., 2011[3]; Johra et al., 2020[4]). However, evidence supporting its disease-preventive effects requires further investigation. Given the expanding literature on the biological activities, therapeutic potential, and delivery strategies of β-carotene, this letter highlights selected recent advances in these areas (Supplementary Table 1).

## Notes

Chanung Park and Jae Kwang Kim contributed equally as first author.

## Declaration

### Acknowledgments

This work was supported by the Rural Development Administration (RDA), Republic of Korea, in 2026, and funded by the National Research Foundation of Korea (NRF) (Project No. RS-2026-25517616).

### Conflict of interest

The authors declare no conflict of interest.

### Artificial Intelligence (AI) - assisted technology

The authors used ChatGPT solely to improve English language, readability, and clarity of text. The authors reviewed and edited AI-assisted output and take responsibility for the content of the manuscript.

## Supplementary Material

Supplementary information
