# Supplementary material for: Recent advances in ß-carotene: Biological activities, therapeutic potential, and delivery strategies
Source: EXCLI J. 2026 Sep 7;25:1458–9. doi: 10.17179/excli2026-9677 (PMC13639177; doi:10.17179/excli2026-9677)
Supplement: Supplementary file 1 [file EXCLI-25-1458-s-001.pdf]

EXCLI Journal 2026;25:1458-1459 – ISSN 1611-2156
Received: July 21, 2026, accepted: August 31, 2026, published: September 07, 2026

Supplementary information to:

Letter to the editor:

RECENT ADVANCES IN Β-CAROTENE: BIOLOGICAL ACTIVITIES, THERAPEUTIC POTENTIAL, AND DELIVERY STRATEGIES

Chanung Park[1†], Jae Kwang Kim[2†], Sang Un Park[1,3*]

[1] Department of Crop Science, Chungnam National University, 99 Daehak-ro, Yuseong-gu, Daejeon 34134, Republic of Korea
[2] Division of Life Sciences and Convergence Research Center for Insect Vectors, College of Life Sciences and Bioengineering, Incheon National University, Incheon 22012, Republic of Korea
[3] Department of Smart Agricultural Systems, Graduate School, Chungnam National University, Daejeon 34134, Republic of Korea

[†] These authors contributed equally to this work.

* **Corresponding author:** Sang Un Park, Department of Crop Science, Chungnam National University, 99 Daehak-ro, Yuseong-gu, Daejeon 34134, Republic of Korea; Tel.: +82-42-821-5730; Fax: +82-42-822-2631; E-mail: supark@cnu.ac.kr

https://dx.doi.org/10.17179/excli2026-9677

This is an Open Access article distributed under the terms of the Creative Commons Attribution License (https://creativecommons.org/licenses/by/4.0/).

Supplementary Table 1: Selected recent studies on the biological, pharmacological and delivery-related applications of β-carotene

Key Findings	Reference
Combined treatment with transcranial static magnetic field stimulation and β-carotene nanoparticles showed marked protection against the behavioural and neurochemical alterations induced by reserpine in the cortex and hippocampus of rats. In this experimental model, the combination exerted antidepressant-like activity.	Khadrawy et al., 2026
An in vitro study of the liposomal co-delivery of β-carotene and doxorubicin in MCF-7 breast cancer cells showed reduced toxicity towards non-cancerous cells compared with doxorubicin alone while preserving anticancer activity. The findings suggest improved therapeutic selectivity in vitro and warrant further evaluation in breast cancer models in vivo.	Elrahman et al., 2026
β-Carotene isolated from the green alga *Westella botryoides* exhibited in vitro antibacterial activity against bacterial strains. Molecular docking suggested favourable interactions with bacterial targets, particularly those of *Staphylococcus aureus*, while in silico analyses indicated a relatively low predicted toxicity profile. These findings support further evaluation of β-carotene as a natural antimicrobial agent.	Alubaidy et al., 2026

EXCLI Journal 2026;25:1458-1459 – ISSN 1611-2156
Received: July 21, 2026, accepted: August 31, 2026, published: September 07, 2026

Key Findings	Reference
This study demonstrated that a Fu brick tea polysaccharide conjugate (FBTPC)–hydroxypropyltrimethyl ammonium chloride chitosan (HACC) complex emulsion exhibited improved emulsion stability and enhanced encapsulation efficiency and protection of β-carotene, in addition to improved intestinal delivery, indicating its potential as a carrier for hydrophobic bioactives.	Zheng et al., 2026
The study showed that 30 min of moderate trypsin hydrolysis produced optimal soybean protein fibril-based Pickering emulsions, which retained β-carotene after stabilisation and 3D printing while improving its stability and bioaccessibility, as well as enhancing fibril conformation and interfacial behaviour.	Wang et al., 2026
The study developed disc-drum emulsion-filled gels with a glycaemic-modulating function based on biodegradable tapioca starch–yeast protein–hydrocolloid materials to serve as β-carotene delivery systems for dysphagia-oriented applications. The compact gel network improved 3D printability, structural integrity, thermal protection, and β-carotene bioaccessibility.	Kong et al., 2026
These results demonstrated improved encapsulation efficiency, structural and oxidative stability, photoprotective capacity, and gastrointestinal bioaccessibility of β-carotene in xanthan gum-coated sunflower oil body emulsions, providing new insights into the use of xanthan gum-coated sunflower oil body emulsions as potential carriers for lipophilic bioactive compounds.	Sheng et al., 2026
β-Carotene also increased lipolysis, activated the forkhead box L2 (FOXL2)–steroidogenic acute regulatory protein (StAR) pathway to enhance estradiol synthesis in granulosa cells, and reduced oxidative stress, thereby supporting follicular development.	Jiang et al., 2026
Ternary complexes of quinoa protein-inulin/chlorogenic acid were found to enhance the interfacial properties, oxidative stability, 3D printability, and β-carotene delivery efficiency of HIPPEs, providing a practical approach for the design of multifunctional emulsions.	Peng et al., 2026
Pickering nanoemulsions stabilised with chitosan nanoparticles significantly improved the stability, environmental resistance, and protection of β-carotene compared with conventional emulsions, indicating their potential application as food-grade delivery platforms for lipophilic bioactive compounds.	Su et al., 2026
Octenyl succinic anhydride-modified myofibrillar protein-stabilised HIPPEs successfully achieved high β-carotene loading, enhanced retention, antioxidant activity, and sustained release of β-carotene after gastrointestinal digestion, suggesting their potential as delivery systems for various bioactive compounds.	Li et al., 2026
β-Carotene blocked DHHC6-mediated S-palmitoylation, restored calcium homeostasis, and alleviated endoplasmic reticulum stress-induced injury. In addition, a reactive oxygen species-responsive β-carotene hydrogel exhibited antibacterial, anti-inflammatory, and bone regenerative effects, supporting its potential application in periodontal therapy.	Huang et al., 2025
Soy lecithin liposomes co-encapsulating β-carotene and doxorubicin exerted synergistic effects by promoting apoptosis and G0/G1 cell-cycle arrest in colorectal cancer cells while reducing doxorubicin-induced cytotoxicity and DNA damage, thereby enhancing co-delivery potential for cancer therapy.	Alhazzaa et al., 2025
In an early colitis-induced colorectal cancer model, sex differences were observed: disease activity index scores increased and serum oestradiol concentrations decreased more markedly in female than in male mice. β-Carotene supplementation significantly reduced disease activity index scores and systemic inflammation in both sexes, although reductions in serum cytokine levels were observed only in females.	Han et al., 2025
Liposomal β-carotene modified with stearyl amine enhanced membrane interactions and increased cytotoxicity and cell-cycle arrest in prostate cancer cells following gamma irradiation, supporting further preclinical evaluation of the formulation.	Abdelrahman et al., 2026

EXCLI Journal 2026;25:1458-1459 – ISSN 1611-2156
Received: July 21, 2026, accepted: August 31, 2026, published: September 07, 2026

Key Findings	Reference
β-Carotene exhibited substantial antifungal and antibiofilm activity by inhibiting the adhesion, hyphal development, and biofilm maturation of drug-resistant *Candida albicans*. These effects improved diabetic wound healing and provide a basis for further therapeutic applications in a diabetic rat model.	Al-Monofy et al., 2025
High dietary β-carotene intake was associated with reduced platelet aggregability among older adults, particularly following stimulation with arachidonic acid and adenosine diphosphate.	Karolczak et al., 2025
Self-assembled nanoparticles derived from sesame protein hydrolysate demonstrated efficient β-carotene delivery with high encapsulation efficiency and sustained release, together with substantially improved bioavailability, indicating their promise as carriers for hydrophobic nutraceuticals.	Liu et al., 2025
The combined use of mung bean protein–glycyrrhizic acid complexes as a versatile emulsifier for HIPPEs enhanced β-carotene loading, stability, bioaccessibility, antioxidant activity, and emulsifying properties, demonstrating their potential for functional food delivery applications.	Yang et al., 2025
Carrot extract rich in β-carotene effectively stained bacterial biofilms, particularly those of Gram-negative bacteria, suggesting its potential as a natural microbial staining agent for the identification of microorganisms.	Câmpelo Lago et al., 2025
Although both types of millet bran nanocellulose-zein nanoparticles showed improved potential for enhancing emulsion stability and β-carotene delivery, the chain-bead-like structures exhibited superior emulsifying performance, digestive stability, and bioaccessibility.	Zhu et al., 2025
The hydrogel combined β-carotene enrichment with antibacterial, antioxidant, self-healing, and sustained-release properties and accelerated the healing of large infected wounds, suggesting its potential for advanced wound dressing applications.	Dan et al., 2025
The stability of bioactive compounds in the extract, sustained antioxidant release, and erythroprotective activity were effectively enhanced by incorporating both *Dunaliella tertiolecta* extract and β-carotene-loaded nanoliposomes, highlighting the potential application of these nanoliposomes in functional foods, nutraceuticals, and biomedical formulations.	García-Morales et al., 2025
β-carotene isolated from *Chlorella vulgaris* may protect human SH-SY5Y cells against apoptosis, oxidative stress, inflammation, and amyloid-β production, suggesting in vitro neuroprotective effects that warrant further validation in animal and clinical studies.	Khan et al., 2025
An alginate hydrogel wound dressing containing silver nanoparticles, β-carotene nanoparticles, and *Centella asiatica* extract exhibited antibacterial and antibiofilm activity against *Staphylococcus aureus* and *Escherichia coli*. Silver-induced cytotoxicity and oxidative stress in human keratinocytes were mitigated by the incorporation of β-carotene and *C. asiatica*, suggesting an improved balance between antimicrobial efficacy and cytocompatibility.	Țăin et al., 2025
In rats recovering from vitamin A deficiency, synthetic β-carotene supplementation reduced the hepatic protein carbonyl levels to values comparable with those in control rats, suggesting attenuation of deficiency-associated hepatic oxidative damage.	Spada et al., 2026
β-carotene, echinenone, and siphonaxanthin reduced nitric oxide production in LPS-stimulated mouse microglia. The effects of β-carotene and echinenone are likely partially mediated through RAR-dependent pathways, whereas those induced by siphonaxanthin are mediated through NF-κB inhibition, suggesting carotenoid-specific modulation of the inflammatory response.	Manabe et al., 2025
Supplementation with β-carotene altered brain tumour cell metabolism through changes in biochemical signatures associated with cytochrome c, lipids, and proteins. The extent and direction of these effects were dependent on cell type, dose, and treatment duration, suggesting a regulatory role in tumour cell metabolism.	Jarczewska et al., 2025

Key Findings	Reference
β-carotene supplementation improved salivary flow in diabetic rats at both 2 and 4 weeks compared to the control treated with saline, suggesting potential to ameliorate diabetes-associated hyposalivation.	Felemban et al., 2025
Higher dietary β-carotene intake was significantly associated with a lower prevalence of colorectal adenomas, and meta-analysis of observational studies supported this inverse association. In contrast, the association of vitamin A and retinol with colorectal adenoma varied depending on dietary context.	Chung et al., 2025
β-Carotene prevented lipopolysaccharide-induced reproductive toxicity in porcine cumulus–oocyte complexes by promoting meiotic maturation, reducing endoplasmic reticulum stress and apoptosis, and improving cumulus–oocyte communication, suggesting a potential fertility-protective role under inflammatory conditions.	Chen et al., 2025
The published findings demonstrate the antioxidant potential of β-carotene in protecting against the non-target organ toxicity of acetamiprid by attenuating oxidative stress and reducing liver and kidney histopathological injury in rats exposed to this insecticide, thereby ameliorating insecticide-induced organ dysfunction.	Awadalla et al., 2025
Maternal β-carotene supplementation may enhance maternal intestinal immune function, increase the transfer of immune mediators such as IgG, lysozyme, and β-defensins to the yolk, and selectively shape the gut microbiota of offspring, thereby promoting growth, intestinal development, immune responses, and overall poultry health.	Wang et al., 2025
Combined supplementation with β-carotene, magnesium, zinc, and metformin improved glycaemic control, reduced oxidative stress, enhanced antioxidant defence, and further protected aged rats with type 2 diabetes against neuropathy, retinopathy, and nephropathy.	Kateel et al., 2025
A β-carotene/β-cyclodextrin inclusion complex demonstrated high encapsulation efficiency, enhanced stability, prolonged release, and retained antioxidant and erythroprotective activities, supporting its potential application as a functional ingredient in food or pharmaceutical formulations.	Puebla-Duarte et al., 2025
In breast cancer cell lines, liposomal β-carotene showed greater in vitro cytotoxicity than free β-carotene. Analyses in MCF-7 cells also indicated pro-apoptotic and DNA-damaging effects, supporting further preclinical evaluation.	Elsayed et al., 2025
β-Carotene supplementation in mice resulted in sex- and tissue-specific modulation of metabolic programming, including enhanced hepatic β-carotene metabolism and reduced gonadal fat deposition in males through interactions involving lipid regulation and oestrogen receptor signalling.	Oh et al., 2025
Oral β-carotene dose-dependently mitigated cardiac fibrosis, collagen deposition, oxidative stress, and associated cardiac dysfunction in rats.	Patil et al., 2025
The hepatic microRNA profile of offspring born to Western diet-fed mothers was altered by early-life β-carotene supplementation in a sex-dependent manner, with a greater number of differentially expressed microRNAs observed in females.	Abrego-Guandique et al., 2024
β-Carotene is a major dietary provitamin A carotenoid that can be converted to retinal and subsequently to retinol or retinoic acid. In particular, retinoic acid may influence lipid metabolism and atherosclerosis through immune modulation involving macrophages and T cells.	Blanco and Amengual, 2024
β-Carotene alleviated LPS-induced endoplasmic reticulum stress and mitochondrial dysfunction in bovine mammary epithelial cells through suppression of ORAI1-mediated calcium signalling.	Meng et al., 2024
At typical dietary exposures, β-carotene appears to be relatively safe and may possess anticancer properties. However, high-dose supplementation in heavy smokers may increase the risk of lung cancer, presumably through oxidative β-carotene metabolites that stimulate cellular proliferation, whereas doses of <15 mg/day may not increase this risk.	Bates et al., 2024

Key Findings	Reference
In male Wistar rats, β-carotene alleviated gentamicin-induced hepatorenal tissue injury and oxidative imbalance, providing preclinical evidence that warrants further investigation.	Sabbagh et al., 2024
Higher dietary β-carotene intake was inversely associated with Parkinson's disease prevalence and risk of all-cause mortality among U.S. adults aged 40 years or older, although causality could not be established.	Su et al., 2024
Pretreatment with β-carotene ameliorated α-amanitin-induced renal injury in rats, with improvements in renal function, reduced oxidative stress, decreased tubular degeneration, modulation of autophagy, and restoration of nitric oxide synthase and aldose reductase activity.	Gezer et al., 2024
β-Carotene exerted renoprotective effects against streptozotocin-induced diabetic nephropathy and histopathological injury by alleviating hyperglycaemia and inflammation, activating the AMPK/SIRT1/autophagy pathway, and inhibiting apoptosis.	El-Marasy et al., 2024
β-Carotene inhibited the proliferation and migration of prostate cancer cells in vitro. Compared with free β-carotene, liposome-encapsulated β-carotene exhibited stronger antiproliferative effects and more pronounced inhibition of epithelial–mesenchymal transition, supporting further preclinical evaluation of the liposomal formulation.	Dulińska-Litewka et al., 2024
Long-term β-carotene supplementation, compared with placebo, did not reduce the incidence of chronic kidney disease among middle-aged and older male physicians, regardless of smoking status, whether chronic kidney disease was defined by self-report or estimated glomerular filtration rate.	Chewcharat et al., 2024
β-carotene-loaded Pickering emulsions without synthetic surfactants were stabilised by food-grade sodium caseinate-lysozyme complex particles, and body-temperature-responsive microcapsules enabled physiological-temperature-dependent release of β-carotene, demonstrating their potential as delivery systems.	Xia et al., 2024
Prospectively assessed moderate intakes of vitamin C, vitamin E, and β-carotene were associated with a lower risk of type 2 diabetes, partly through reduced insulin resistance, although randomised controlled trials have not demonstrated a clear protective effect of antioxidant supplementation.	Lampousi et al., 2024
β-Carotene enhanced the anticancer effects of oxaliplatin against colorectal cancer by inducing apoptosis and G2/M cell-cycle arrest, reducing cancer stemness and drug resistance, while exhibiting low toxicity towards normal organoids.	Lee et al., 2024
In mice, β-carotene promoted the resolution of atherosclerosis through BCO1-dependent vitamin A production. This effect was associated with increased endogenous regulatory T cells within atherosclerotic plaques, indicating a potential immune-mediated mechanism of cardiovascular protection.	Pinos et al., 2024
β-Carotene attenuated ethanol-induced pancreatic injury in mice, as evidenced by improvements in pancreatic enzyme levels, fibrosis scores, and islet/acinar cell morphology, suggesting a protective effect of β-carotene in this mouse model.	Sandoval et al., 2024

REFERENCES

Abdelrahman M, Shafaa MW, Abdelrahman H, Ibrahim M. Positively charged liposomal β-carotene enhances irradiation-induced cytotoxicity against prostate carcinoma cells: an in-vitro study. BMC cancer. 2026;26:6.
https://doi.org/10.1186/s12885-025-15244-z

Abrego-Guandique DM, Galmés S, García-Rodríguez A, Cannataro R, Caroleo MC, Ribot J, et al. β-Carotene impacts the liver MicroRNA profile in a sex-specific manner in mouse offspring of Western diet-fed mothers: results from microarray analysis by direct hybridization. International journal of molecular sciences. 2024;25:12899.
https://doi.org/10.3390/ijms252312899

Al-Monofy KB, Abdelaziz AA, Abo-Kamar AM, Al-Madboly LA, Farghali MH. The dual antifungal and antibiofilm activities of β-carotene against multidrug-resistant Candida albicans induce wound healing in a diabetic rat model: an in vitro and in vivo study. BMC microbiology. 2025;25:747.
https://doi.org/10.1186/s12866-025-04447-w

Alhazzaa DM, Shafaa MW, Elabed S, Shafaa AM, Omran MM. Liposomal co-delivery of β-carotene and doxorubicin for enhanced colorectal-cancer therapy. Scientific Reports. 2025;15:44148.
https://doi.org/10.1038/s41598-025-27935-5

Alubaidy HA, Al-Bdeery AH, Owda ZAA, Alghanmi HA, Ghotekar S, Jabir MS. In Silico and In-vitro assessment of β carotene extracted from Westella botryoides for fighting multidrug resistant bacterial strains. International Microbiology. 2026;29:579–599.
https://doi.org/10.1007/s10123-025-00767-z

Awadalla EA, Amin YA, Ali RA, Gbr SA, Gelany WI, Nour AH. Co-treatment of β-carotene with acetamiprid provides protection against acetamiprid induced hepatic and renal toxicity via modulation of the antioxidant system. BMC Pharmacology and Toxicology. 2025;26:122.
https://doi.org/10.1186/s40360-025-00953-9

Bates CA, Vincent MJ, Buerger AN, Santamaria AB, Maier A, Jack M. Investigating the relationship between β-carotene intake from diet and supplements, smoking, and lung cancer risk. Food and Chemical Toxicology. 2024;194:115104.
https://doi.org/10.1016/j.fct.2024.115104

Blanco A, Amengual J. Immunometabolic effects of β-carotene and vitamin A in atherogenesis. Immunometabolism. 2024;6:e00051.
https://doi.org/10.1097/IN9.0000000000000051

Câmpelo Lago E, Vasconcelos AG, Rodrigues de Araújo-Nobre A, Salmen Espindola L, Quelemes PV, Arcanjo DDR, et al. Natural pigments as eco-friendly staining agents: evaluation of β-carotene-rich carrot and betanin-rich beetroot extracts for bacterial biofilm visualisation. Natural Product Research. 2025:1–5.
https://doi.org/10.1080/14786419.2025.2584437

Chen M, Li J, He Y, Wang W, Ju S, Li Q. Protective effect of β-carotene against lipopolysaccharide-induced meiotic maturation defects in porcine cumulus-oocytes complexes. Animal Reproduction Science. 2025;279:107947.
https://doi.org/10.1016/j.anireprosci.2025.107947

Chewcharat A, Chewcharat P, Rexrode KM, Glynn RJ, Buring JE, Gaziano JM, et al. The effect of randomized Beta-carotene supplementation on CKD in men. Kidney International Reports. 2024;9:1633–40.
https://doi.org/10.1016/j.ekir.2024.04.001

Chung J, Kang J, Kim SH, Jung MK, Kim DH, Song HJ, et al. Dietary β-Carotene, Vitamin A, and Retinol Intake and Prevalence of Colorectal Adenoma: A Cross-Sectional Study and Meta-Analysis. Gut and liver. 2025;19:845.
https://doi.org/10.5009/gnl250104

Dan A, Singh H, Darban Z, Yadav I, Munaware J, Shah SA, et al. Therapeutic β-carotene-loaded inulin-based multifunctional hydrogel for full-thickness skin wounds with polymicrobial infections. Carbohydrate Polymers. 2025;370:124381.
https://doi.org/10.1016/j.carbpol.2025.124381

Dulińska-Litewka J, Dykas K, Boznański S, Hałubiec P, Kaczor-Kamińska M, Zagajewski J, et al. The Influence of β-carotene and its liposomal form on the expression of EMT markers and androgen-dependent pathways in different prostate cell lines. Antioxidants. 2024;13:902.
https://doi.org/10.3390/antiox13080902

El-Marasy SA, Farouk H, Khattab MS, Moustafa PE. Beta-carotene ameliorates diabetic nephropathy in rats: involvement of AMPK/SIRT1/autophagy pathway. Immunopharmacology and Immunotoxicology. 2024; 46:763–72.
https://doi.org/10.1080/08923973.2024.2402347

Elrahman HMA, Shafaa MW, Masoomi M, Ibrahim M, Elbaset MA, Ibrahim AM. Biophysical and biological evaluation of free and liposomal doxorubicin and β-carotene reveals enhanced therapeutic selectivity in MCF-7 breast cancer cells. Journal of Liposome Research. 2026:1–16.
https://doi.org/10.1080/08982104.2026.2671264

Elsayed MH, Shafaa MW, Abdalla MS, El-Khadragy MF, Moneim AEA, Ramadan SS. Antitumor assessment of liposomal beta-carotene with tamoxifen against breast carcinoma cell line: an in vitro study. Biomolecules. 2025;15:486.
https://doi.org/10.3390/biom15040486

Felemban MF, Bin Mubayrik AF, Alotaibi O. The Effect of Vitamin E, B-Carotene, and Acidophilus Probiotics on The Salivary Gland Flow Rate in Streptozotocin-Induced Diabetes in Rats: An in-vivo Study. Clinical, Cosmetic and Investigational Dentistry. 2025;17:335–47.
https://doi.org/10.2147/CCIDE.S528114

García-Morales J, González-Vega RI, Fimbres-Olivarría D, Bernal-Mercado AT, Auobourg-Martínez SP, López-Gastélum KA, et al. Nanoencapsulated Dunaliella tertiolecta Extract and β-Carotene in Liposomal Carriers: Antioxidant and Erythroprotective Potential Through Sustained-Release Systems. Molecules. 2025;30:3924.
https://doi.org/10.3390/molecules30193924

Gezer A, Üstündağ H, Sarı EK, Bedir G, Gür C, Mendil AS, et al. β-carotene protects against α-amanitin nephrotoxicity via modulation of oxidative, autophagic, nitric oxide signaling, and polyol pathways in rat kidneys. Food and Chemical Toxicology. 2024;193:115040.
https://doi.org/10.1016/j.fct.2024.115040

Han H, Park S, Han S, Kim Y. Sex differences of β-carotene effects in azoxymethane/dextran sulfate sodium-induced early colorectal cancer mice. Nutrition Research and Practice. 2025;19:891–903.
https://doi.org/10.4162/nrp.2025.19.6.891

Huang S, Li M, Cheng Z, He M, Deng J, Zhang D, et al. β-Carotene liposome-based responsive hydrogel inhibits palmitoylation to restore intracellular calcium homeostasis for effective periodontitis treatment. Journal of Nanobiotechnology. 2025;24:107.
https://doi.org/10.1186/s12951-025-03973-z

Jarczewska K, Kopeć M, Abramczyk H, Surmacki JM. Raman spectroscopy and imaging reveal the effect of β-carotene supplementation on brain cancer cells. Biochemistry. 2025;64:3745–59.
https://doi.org/10.1021/acs.biochem.5c00231

Jiang J, Chen L, Ma W, Wen T, Liu R, Chu G, et al. Dietary β-carotene improves the ovary development and antioxidant capacity of replacement gilts. Journal of Animal Science and Biotechnology. 2026;17:23.
https://doi.org/10.1186/s40104-025-01342-2

Karolczak K, Guligowska A, Sołtysik BK, Kostanek J, Kostka T, Watala C. Estimated Amounts of β-Carotene, Vitamin B6, Riboflavin and Niacin in the Daily Diet of Older Subjects Associate Negatively with ADP-Induced Aggregation of Blood Platelets Independently of Cardiovascular Risk Factors. Nutrients. 2025;17:3461.
https://doi.org/10.3390/nu17213461

Kateel R, Kashyap NN, Reddy SK, Shetty S, Kumari MK, Ullal SD, et al. Chronic β-carotene, magnesium, and zinc supplementation together with metformin attenuates diabetes-related complications in aged rats. Clinical Nutrition. 2025;50:183–97.
https://doi.org/10.1016/j.clnu.2025.05.004

Khadrawy YA, Mohammed HS, Hosny EN, Elkholy NS, Aboul Ezz HS. Antidepressant effects of transcranial static magnetic field stimulation and β-carotene nanoparticles in a rat model of depression. Progress in Neuro-Psychopharmacology and Biological Psychiatry. 2026;147:111753.
https://doi.org/10.1016/j.pnpbp.2026.111753

Khan MI, Jeong ES, Tasreen G, Khan MZ, Shin JH, Kim JD. The therapeutic potential of beta-carotene against neuroinflammation and amyloid beta in SH-SY5Y cells. Scientific Reports. 2025;15:33803.
https://doi.org/10.1038/s41598-025-00964-w

Kong Y, Chen J, Lu K, Huang Q. Hydrocolloid mediated reconstruction and functionality of tapioca starch–yeast protein emulsion-filled gels: Toward β-carotene delivery and dysphagia-friendly foods of 3D printing. Food Chemistry. 2026;509:148555.
https://doi.org/10.1016/j.foodchem.2026.148555

Lampousi AM, Lundberg T, Löfvenborg JE, Carlsson S. Vitamins C, E, and β-Carotene and Risk of Type 2 Diabetes: A Systematic Review and Meta-Analysis. Advances in Nutrition. 2024;15:100211.
https://doi.org/10.1016/j.advnut.2024.100211

Lee J, Heo SC, Kim Y. Combination of oxaliplatin and β-carotene suppresses colorectal cancer by regulating cell cycle, apoptosis, and cancer stemness in vitro. Nutrition Research and Practice. 2024;18:62–77.
https://doi.org/10.4162/nrp.2024.18.1.62

Li F, Huang L, Yuan C, Dai M, Yang X, Lang Y. High internal phase Pickering emulsion stabilized by succinylation of myofibrillar protein for β-carotene delivery. Food Chemistry. 2026;498:147188.
https://doi.org/10.1016/j.foodchem.2025.147188

Liu Y, Zhu Z, Ai X, Pan F, Tian W, Zhao L, et al. Self-assembled peptides derived from sesame meal as novel carriers for β-carotene with enhanced bioavailability: A study integrating experimental and computational perspectives. Food Chemistry. 2025;497:147015.
https://doi.org/10.1016/j.foodchem.2025.147015

Manabe Y, Ueda M, Sugawara T. β-Carotene Suppresses Lipopolysaccharide-induced Nitric Oxide Production in Microglia via Retinoic Acid Receptor-dependent Mechanisms. Journal of Oleo Science. 2025;74:845–57.
https://doi.org/10.5650/jos.ess25072

Meng M, Li X, Zhou S, Shi X, Shen X, Chang G. β-Carotene Ameliorates LPS-Induced Endoplasmic Reticulum Stress and Mitochondrial Disorder by Targeting ORAI1 in Bovine Mammary Epithelial Cells. Journal of Agricultural and Food Chemistry. 2024;72:26733–45.
https://doi.org/10.1021/acs.jafc.4c06875

Oh Y, Kim J, Park YJ, Kim Y. Male-Specific Effects of β-Carotene Supplementation on Lipid Metabolism in the Liver and Gonadal Adipose Tissue of Healthy Mice. Molecules. 2025;30:909.
https://doi.org/10.3390/molecules30040909

Patil N, Patil VS, Punase N, Mapare G, Bhatt S, Patil CR. Comparative Efficacy of β-Carotene and Losartan Against Isoproterenol-Induced Cardiac Fibrosis: An Experimental and Computational Studies. Journal of the American Nutrition Association. 2025;44:529–44.
https://doi.org/10.1080/27697061.2025.2461217

Peng X, Wang K, Ji Y, Jin Y, Shen X. Improved oxidation stability, 3D printing, and β-carotene delivery performance of high internal phase Pickering emulsions using quinoa protein-inulin/chlorogenic acid ternary complexes. Food Research International. 2026;226:118178.
https://doi.org/10.1016/j.foodres.2025.118178

Pinos I, Coronel J, Albakri A, Blanco A, McQueen P, Molina D, et al. β-Carotene accelerates the resolution of atherosclerosis in mice. eLife. 2024;12:RP87430.
https://doi.org/10.7554/elife.87430

Puebla-Duarte AL, Bernal-Mercado AT, Santos-Sauceda I, Acosta-Elias M, Fernández-Quiroz D, Burruel-Ibarra SE, et al. The Characterization and Antioxidant and Erythroprotective Effects of β-Carotene Complexed in β-Cyclodextrin. International Journal of Molecular Sciences. 2025;26:3902.
https://doi.org/10.3390/ijms26083902

Sabbagh S, Rayatpishe P, Goudarzi M, Behvandi MM, Norouzirad R. Protective effect of beta-carotene on hepato-nephrotoxicity of gentamicin in male Wistar rats. Tissue and Cell. 2024;91:102613.
https://doi.org/10.1016/j.tice.2024.102613

Sandoval C, Vera A, Birditt K, Godoy K, Carmine F, Caamaño J, et al. β-Carotene Supplementation Improves Pancreas Function during Moderate Ethanol Consumption: Initial Characterization from a Morphological Overview. International Journal of Molecular Sciences. 2024;25:1219.
https://doi.org/10.3390/ijms25021219

Sheng X, Zaaboul F, Chen L, Chen L, Yang R, Zhao L. Xanthan Gum-Stabilized Sunflower Oil Body Emulsions for β-Carotene Delivery: Preparation, Stability, and Digestion Behavior. Foods. 2026;15:567.
https://doi.org/10.3390/foods15030567

Spada ECL, Dahmer DSV, Barboza AM, Silva WBD, Neto EWA, Boaventura MF, et al. β-carotene and fatty acid composition of Attalea phalerata oil and its effect on metabolism as the sole source of vitamin A precursors in deficient rats. Nutrition and Health. 2026;32:1331–9.
https://doi.org/10.1177/02601060251375505

Su J, Liu L, Wang R, Li C, Wang Z, Xu Q, et al. Association between dietary β-carotene intake with Parkinson's disease and all-cause mortality among American adults aged 40 and older (NHANES 2001–2018). Frontiers in Nutrition. 2024;11:1430605.
https://doi.org/10.3389/fnut.2024.1430605

Su S, Lu J, Yin X, Wang M, Wang F, Pang Y. Encapsulation of β-carotene in chitosan Pickering nanoemulsion: Effect of process parameters and their stability. Food Chemistry. 2026;500:147505.
https://doi.org/10.1016/j.foodchem.2025.147505

Țăin AE, Bîrcă AC, Nedelcu MS, Holban AM, Niculescu AG, Grumezescu AM, et al. Cinnamon-Mediated Silver Nanoparticles and Beta-Carotene Nanocarriers in Alginate Dressings for Wound Healing Applications. Gels. 2025;11:738.
https://doi.org/10.3390/gels11090738

Wang T, Wang G, Wu M, Lan H, Liu J, Gong H, et al. Maternal β-carotene supplementation improves offspring growth, development, immunity, and intestinal microbiota in chickens via immune-mediated and microbial-mediated maternal effects. Scientific Reports. 2025;15:19149.
https://doi.org/10.1038/s41598-025-03450-5

Wang Y, Zhao M, Tan H, Wu X, Wu W. Improvement of β-carotene stability and bioaccessibility by soybean protein fibrils-high internal phase Pickering emulsions: Trypsin hydrolysis contribution. International Journal of Biological Macromolecules. 2026;352:151207. https://doi.org/10.1016/j.ijbiomac.2026.151207

Xia C, Xu Z, Xu M, Zhang C, Xu B, Liu B, et al. Body temperature responsive capsules templated from Pickering emulsion for thermally triggered release of β-carotene. International Journal of Biological Macromolecules. 2024;266:130940. https://doi.org/10.1016/j.ijbiomac.2024.130940

Yang H, Wang B, Wei Y, Gu C, Wu X. High internal-phase pickering emulsions stabilized by mung bean protein–glycyrrhizin complexes for β-carotene delivery. Food Chemistry. 2025;497:147036. https://doi.org/10.1016/j.foodchem.2025.147036

Zheng W, Gao Y, Xu Q, Zhang J, Deng W, Chen Y. Self-assembly of Fu brick tea polysaccharide conjugate and chitosan quaternary ammonium salt for the synergistic stabilization of β-carotene-loaded complex emulsions with enhanced stability and bioaccessibility. International Journal of Biological Macromolecules. 2026;356:151476. https://doi.org/10.1016/j.ijbiomac.2026.151476

Zhu Y, Wei Z, Shi Q, Dan Q, Li M, Yu X, et al. Morphology-regulated millet bran nanocellulose-zein nanoparticles complexes: stabilization of Pickering emulsions and β-carotene delivery. Food Chemistry: X. 2025;31:103136. https://doi.org/10.1016/j.fochx.2025.103136
